# ZnO-modified carbon paste electrode for electrochemical sensing of dopamine in the presence of tyrosine

**DOI:** 10.5599/admet.3010

**Published:** 2025-10-21

**Authors:** Ali Obaid Imarah, Nada Hasan, Mustafa G. Alabbasi

**Affiliations:** 1 Department of Chemical Engineering, College of Engineering, University of Babylon, Babylon Iraq; 2 Department of Basic Science, College of Dentistry, Al-Mustaqbal University, Babylon, Hillah, 51001, Iraq; 3 Department of Pharmacology and Toxicology, College of Pharmacy, University of Alkafeel, Iraq

**Keywords:** ZnO nanoparticles, voltammetry, Alzheimer's disease, Parkinson's disease, chronoamperometry

## Abstract

**Background and purpose:**

Dopamine, 3,4-dihydroxyphenylalanine, functions as a catecholamine neurotransmitter in the brain, sending messages to other neurons to regulate information transmission to other areas of the brain, govern movement, and alter brain activity. Tyrosine undergoes an enzymatic process in the pharmaceutical industry to produce dopamine. Thus, it is crucial to measure both tyrosine and dopamine in bodily fluids simultaneously.

**Experimental approach:**

In this work, we demonstrate the production of ZnO nanoparticles using a straightforward solvothermal technique. A straightforward, quick, and sensitive electrochemical sensing platform for dopamine detection was then created using the produced ZnO nanoparticles.

**Key results:**

Cyclic voltammetry comparison revealed that the ZnO/carbon paste electrode considerably enhanced the dopamine oxidation process compared to the unmodified carbon paste electrode (CPE). With a low detection limit of 0.003 μM, the ZnO/CPE sensor's linear response for voltammetric dopamine determination was found to be between 0.01 and 480.0 μM.

**Conclusion:**

The modified CPE effectively demonstrates its great accuracy in tyrosine-induced dopamine detection.

## Introduction

A neurodegenerative condition in the central nervous system produces Parkinson's disease (PD), which manifests as a compromised motor system. The disease's most typical symptoms are delayed movement, postural instability, poor balance, stiff arms and legs, and resting tremor of the hands and jaw. Non-motor symptoms (NMSs) include depression, dementia, sleep issues, and olfactory disorders, which typically manifest before motor dysfunction [1,2].

Although the primary aetiology of Parkinson's disease is still unknown, it is known that environmental, genetic, and age variables all have an impact on the illness's occurrence. Dopamine deficiency results from the death of dopaminergic neurons in the midbrain region. Dopamine has not been shown to alleviate the symptoms yet. As a dopamine replacement, levodopa (L-DOPA) is the standard therapy in the early stages [3]. The molecular name for dopamine (DA), an organic molecule, is 3,4-dihydroxyphenylalanine. It functions as a catecholamine neurotransmitter in the brain, sending messages to other neurons to regulate information transmission to other areas of the brain, govern movement, and alter brain activity [4,5].

Dopamine is present in the brain's caudate nucleus at a greater concentration of 50 nmol g^-1^ and in extracellular fluids at a lower value of 0.01 to 1.00 ìM. Euphoria may be caused by an overabundance of dopamine [6,7]. Alzheimer's, Parkinson's, and Huntington's disease are among the severe neurological conditions brought on by disruptions in dopamine levels [8,9]. Tyrosine (Ty) is enzymatically processed in the pharmaceutical industry to produce dopamine [10]. Thus, it is crucial to measure both tyrosine and dopamine in bodily fluids simultaneously [11].

Tyrosine maintains a favourable nitrogen balance in the human body in addition to being a key component of proteins [12]. Dairy products, wheat, eggs, fish, meats, and supplements can help compensate for the lack of tyrosine in vegetables [13]. It is also utilized as a mood enhancer, hunger suppressant, and hormone growth stimulant since it alters neurotransmitters. Additionally, it is proven to have antioxidant properties [14]. In addition to psychiatric issues, low tyrosine levels induce alkaptonuria and albinism. Conversely, a high tyrosine level increases the likelihood of sister chromatid exchanges and causes Parkinson's disease [15].

Spectrophotometry [16], GC [17], LC [18], HPLC [19], and chemiluminescence are the most widely used techniques for determining dopamine and tyrosine. However, these methods have some limitations that restrict their use, such as costly equipment, interferences, limited sensitivity, and low selectivity [21].

Due to their strong electrochemical activity, electrochemical techniques for simultaneous dopamine and tyrosine detection offer the benefits of simplicity, speed, low costs and excellent sensitivities and selectivities [22,23].

Nanomaterials have been introduced to improve the electrochemical performance. With several applications in biosensors, the fabrication of customized electrodes using nanoparticles is still an unexplored field of study [23,24]. Due to their ease of use, moderate accuracy and precision, affordability, and speed, electrochemical methods have been developed for pharmaceutical analysis. Compared to other methods, the electroanalytical method is less sensitive to matrix effects; hence, there is no need for derivatizations or laborious extraction procedures [25,26].

Due to their low residual currents and noise, ease of manufacture, broad anodic and cathodic potential ranges, rapid surface renewal, and affordability, carbon paste electrodes (CPEs) are frequently used for electrochemical assessments of various biological and pharmacological species. To improve the sensitivity, selectivity, and speed of determinations, it is also simple to create chemically modified electrodes (CMEs) by mixing various materials with the bulk of CPEs [15-29].

ZnO is one of the most attractive semiconductors due to its hexagonal wurtzite structure, broad band gap, high exciton binding energy and numerous applications in sensors, optoelectronic devices, photonic detectors, polarized light-emitting devices, catalysis, photovoltaics, and more. Many one-dimensional morphologies, including nanobelts [30], nanorods [31], and nanowires [32], are available for ZnO, which may also be manufactured in a range of single crystal forms, including thin films. The creation of sensors based on ZnO nanomaterial is greatly aided by these nanostructures' remarkable mechanical stability and large surface-to-volume ratios, which also enable electrode modification as a feasible alternative [34].

This study describes the development of a novel electrode composed of ZnO nanostructures (ZnO/CPE) and examines its effectiveness in measuring dopamine. Additionally, we evaluate the modified electrode's analytical performance for measuring dopamine in the presence of tyrosine.

## Experimental

### Apparatus and chemical

Electrochemical tests were conducted utilizing an Autolab PGSTAT 302N, controlled by GPES software, which allowed setting and monitoring test conditions. The SPE system (DropSens, DRP-110, Spain) included three electrodes: a working electrode based on graphite, a silver pseudo-reference electrode, and a graphite counter electrode. All chemicals (including analytical grade dopamine and tyrosine) were sourced through Merck (Darmstadt, Germany). Phosphate buffer solutions were prepared using H_3_PO_4_ and the corresponding salt.

### Synthesis of ZnO nanostructures

The following procedure was used to synthesize ZnO nanostructures [35]: 10 mL of ethanol was used to prepare a 0.2 M Zn(NO_3_)_2_·6H_2_O solution. After 40 minutes of magnetic stirring, 70 mL of NaOH 0.5 M was added to the solution above. For 12 hours, the prepared solution was placed in an autoclave and maintained at 180 °C. The precipitates were centrifuged, cleaned with ethanol and deionized water, and then allowed to settle to room temperature before being dried overnight at 60 °C.

### Preparation of the electrodes

Using a mortar and pestle, 0.04 g of ZnO nanostructure and 0.96 g of graphite powder were manually mixed to create the ZnO/CPE. After adding paraffin oil, the mixture was stirred for 20 minutes to create an evenly moist paste. The end of a glass tube was then filled with the paste. The electrical contacts were made by inserting a copper wire into the CP.

### Preparation of real samples

To make 10 mL, 1 mL of a dopamine ampoule was diluted with PBS. A varying volume of this solution was then put into volumetric flasks and diluted many times with PBS until it reached 25 mL. Lastly, the suggested electrode used the conventional addition approach to measure the dopamine content.

Samples of urine were promptly placed in a refrigerator upon collection. For fifteen minutes, 10 mL of the samples was centrifuged at 2,000 rpm. After that, a 0.45 μm filter was used to filter the supernatant. Following that, varying amounts of this solution were added to volumetric flasks and diluted with PBS to a final volume of 25 mL. Tyrosine and dopamine were added to the diluted urine samples at varying concentrations. Lastly, the suggested electrode used the conventional addition technique to analyse the dopamine and tyrosine contents.

## Result and discussion

### Electrochemical behaviour of the analyte on the ZnO / carbon paste electrode

Since the pH value affects how dopamine behaves electrochemically, the pH was adjusted prior to the study to obtain the most accurate findings. Accordingly, dopamine electrooxidation at the surface of the modified electrode was carried out at different pH values (2.0 to 9.0), with the best results achieved at pH 7.

**Figure 1.**
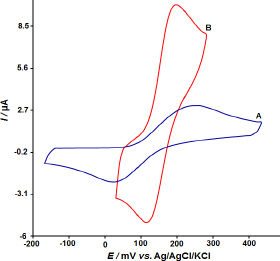
CV response of dopamine at (a) bare CPE and (b) ZnO/CPE

Cyclic voltammograms of 100.0 μM dopamine on an unmodified CPE and the ZnO/CPE) are shown in Figure 1.

The voltammograms clearly show that the ZnO/CPE produces higher dopamine oxidation currents at 200 mV, which is around 50 mV lower than the maximum achieved with the unmodified CPE. Furthermore, it is evident that the peak currents in the ZnO/CPE studies are significantly greater, demonstrating the significant improvement in the modified electrode's electrochemical behaviour for dopamine sensing.

### Effect of scan rate

**Figure 2.**
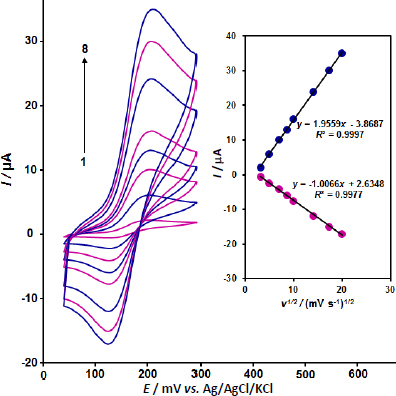
CV curves of 100.0 μM dopamine at different scan rates (10 to 400 mV s^-1^) on ZnO/CPE (1 to 8: 10, 25, 50, 75, 100, 200, 300 and 400 mV s^-^). Inset: Plot of scan rate square root versus dopamine peak current

The impact of potential scan rates on dopamine currents is seen in Figure 2. As demonstrated, higher peak currents result from an increase in scan rate. Furthermore, the *I*_p_ *vs. v^1/2^* graph for dopamine was linear, indicating that diffusion controls the oxidation processes of dopamine.

### Chronoamperometric measurements

**Figure 3.**
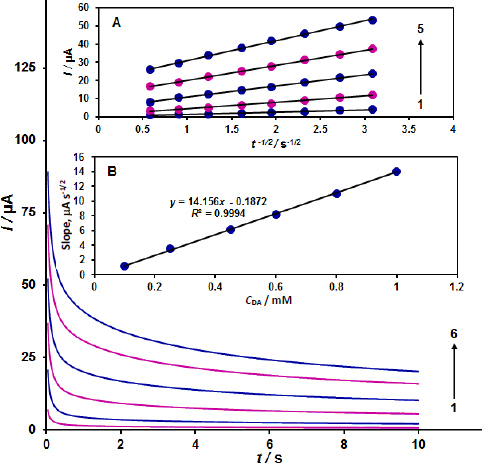
The chronoamperograms obtained on ZnO/CPE at different dopamine concentrations; Note: 1 to 6: 0.1, 0.25, 0.45, 0.6, 0.8 and 1.0 mM of dopamine. Inset A - plot of *I* versus *t*^-1/2^ based on chronoamperograms. Inset B - slope plot of straight-line versus dopamine concentration



The ZnO/CPE was used to perform chronoamperometric studies of the dopamine sample at 0.25 V. Figure 3 shows the result for the various dopamine samples. To describe the time dependence of current in the diffusion-controlled process, the Cottrell Equation (1) is usually used:

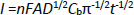

(1)


The best-fitting *I* vs. *t*^−1/2^ plots were created for various dopamine samples based on experimental data. The slopes of the generated straight lines were then plotted against the dopamine. *D* values for dopamine were found to be 2.08×10^-6^ cm^2^ s^-1^, based on the Cottrell Equation (1) and the corresponding slopes.

### Calibration curve

**Figure 4.**
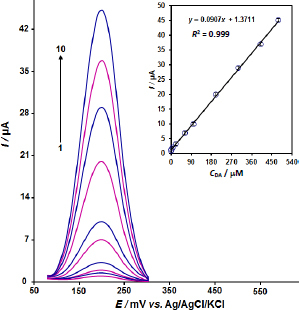
DPV response of dopamine at ZnO/CPE in the concentration range 0.1 to 900.0 μM in PBS (0.1 M, pH 7.0); 1 to10 refers to 0.01, 0.8, 5.0, 20.0, 60.0, 100.0, 200.0, 300.0, 400.0 and 480.0 μM; inset: The calibration curve of DPV peaks against concentration of dopamine

The oxidation peak currents of dopamine and tyrosine were measured in aqueous media of the two target compounds using a ZnO/CPE. Because of its higher sensitivity and analytical ease, DPV was performed using the electrode to measure several concentration levels of the analyte. Dopamine exhibited a direct linear relationship between peak current and concentration from 0.01 to 480.0 μM, as shown in Figure 4, which demonstrated an excellent correlation factor (*R*^2^ = 0.9995) with a low LOD of 0.05 μM. Likewise, tyrosine exhibited a linear calibration plot over the range of 0.01 to 480.0 μM with a LOD of 0.003 μM (3*σ*) (not shown).

### Simultaneous determination of dopamine and tyrosine

**Figure 5.**
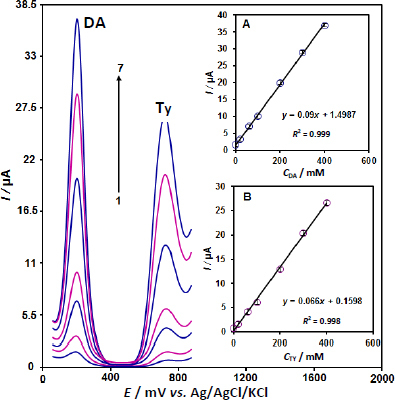
DPVs of ZnO/CPE in different concentrations of dopamine and tyrosine (1 to 7: 1.0 + 1.0, 20.0 + 20.0, 60.0 + 60.0, 100.0 + 100.0, 200.0 + 200.0, 300.0 + 300.0 and 400.0 + 400.0 μM dopamine and tyrosine). Inset A: Plot of the *I* as a function of dopamine concentration. Inset B: Plot of *I* as a function of tyrosine concentration

Using unmodified electrodes, the electrochemical detection of dopamine is challenging due to the overlapping oxidation potential of dopamine and tyrosine. To address this issue, differential pulse voltammograms (Figure 5) were obtained for the ZnO/CPE using various analyte concentrations. The modified electrode was able to distinguish two separate oxidation peaks for the analytes at 200 mV for dopamine and 725 mV for tyrosine, leading to simultaneous detection without any visible interference.

### Sample analysis

The effectiveness of the developed method was investigated for the determination of dopamine and tyrosine in pharmaceutical samples and human urine. The results are presented in Table 1. The results showed a high recovery percentage for both analytes, confirming the effectiveness, precision, and reproducibility of the method based on the low relative standard deviation values.

**Table 1.** Determining dopamine and tyrosine in pharmaceutical ampoules and human urine (*n*=5)

Sample	Amount, μM				Recovery, %		RSD, %	
	Spiked		Found					
	Dopamine	Tyrosine	Dopamine	Tyrosine	Dopamine	Tyrosine	Dopamine	Tyrosine
Dopamine injection	0	0	2.9	-	-	-	3.3	-
	2.0	5.0	5.0	4.9	102.0	98.0	1.9	3.0
	3.0	7.0	5.8	7.3	98.3	104.3	2.8	2.1
	4.0	9.0	6.7	8.9	97.1	98.9	2.2	2.9
	5.0	11.0	8.0	10.9	101.3	99.1	2.6	1.8
Urine	0	0	-	-	-	-	-	-
	5.5	5.0	5.4	5.1	98.1	102.0	3.1	2.0
	6.5	6.0	6.6	5.8	101.5	96.7	2.1	1.7
	7.5	7.0	7.4	7.1	98.7	101.4	1.9	3.5
	8.5	8.0	8.8	7.9	103.5	98.7	2.5	3.0

## Conclusions

The results confirmed the suitability of the ZnO/CPE electrode for the concurrent quantification of dopamine and tyrosine. The observed negative shift in anodic peak potential revealed its electrocatalytic function in promoting the oxidation of both analytes. Owing to the wide peak-to-peak separations, the electrode provided excellent sensitivity for dual detection. Studies on the effect of scan rate indicated that the oxidation mechanism proceeds under diffusion control. With a low LOD of 0.003 μM, the ZnO/CPE sensors exhibit a linear response for voltammetric dopamine determination between 0.01 and 480.0 μM. Key attributes of this sensor include high analytical sensitivity, very low detection thresholds, reliable performance, and low fabrication cost. Collectively, these merits highlight the electrode as an effective and innovative platform for monitoring dopamine and tyrosine in practical sample matrices.
